# Complete model-free sliding mode control (CMFSMC)

**DOI:** 10.1038/s41598-021-01871-6

**Published:** 2021-11-19

**Authors:** Quanmin Zhu

**Affiliations:** Department of Engineering Design and Mathematics, University of the West of England, Frenchy Campus, Coldharbour Lane, Bristol, BS16 1QY UK

**Keywords:** Engineering, Mathematics and computing

## Abstract

This study presents a complete model-free sliding mode control (CMFSMC) framework for the control of continuous-time non-affine nonlinear dynamic systems with unknown models. The novelty lies in the introduction of two equalities to assign the derivative of the sliding functions, which generally bridges the designs of those model-based SMC and model-free SMC. The study includes a double SMC (DSMC) design, state observer design, and desired reference state vector design (whole system performance), which all do not require plant nominal models. The preconditions required in the CMFSMC are the plant dynamic order and the boundedness of plant and disturbances. U-model based control (U-control) is incorporated to configure the whole control system, that is (1) taking model-free double SMC as a robust dynamic inverter to cancel simultaneously both nonlinearity and dynamics of the underlying plants, (2) taking a model-free state observer to estimate the state vector, (3) taking invariant controller to specify the whole control system performance in a linear output feedback control and to provide desired reference state vector. The related properties are studied to support the concept/configuration development and the analytical formulations. Simulated case studies demonstrate the developed framework and show off the transparent design procedure for applications and expansions.

## Introduction

### Model-free sliding mode control

Parallel to model-based control, model-free control, or popularly named as data-driven control, has attracted lager cohort research efforts, typically from data-driven control^1^, model-free control^2^, active disturbance rejection control (ADRC)^3^, adaptive dynamic programming^4^, etc. All the model-free approaches are based on the belief that the modelling process is expensive, time-consuming and inaccurate. For the particular interest of the study, model-free sliding model control (MFSMC), some of the related research has been reported. A representative research publication domain has been in the discrete-time approaches, which has proposed a class of MFSMC with control input difference formulation plus an ad-hoc system formation^5^. The research group has expanded this approach to MIMO systems^6^, which follows the kernel foundation that the control system relies on estimating the control input difference to stabilise the systems. The other MFSMC approach^7^ is using a first-order system as foundation to assign PID controllers plus switching controls, which also provides low-pass filter for estimating the first-order derivative of the controller output and disturbance estimator to facilitate the MFSMC design. Another work^8^ has presented a work on model-free sliding mode control, theory and application, which is based on discrete time formulation again from estimating existing underlying system behaviour, controller optimisation, to sliding mode design. Even claimed as a model free control method, the approach actually uses an online model estimated from the measurement for SMC design. In addition, SMC technique is still expanding quickly to a wide range of dynamic systems, such as a latest leading work^9^ devoted to networked control systems with time delay, using auxiliary matrix-based formulations. No doubt such new approaches will be solid foundation for expansion to model free SMC in the follow up studies.

### State observer

It has been observed that the high cost of the actual equipment, the availability/limitation of sensors and other factors, system state components could not be obtained fully directly from measurement even with noise. For many application domains, such as control system analysis and synthesis, fault detection systems, and various issues in signal processing and identifications, state observer/reconstruction has been generally widely adopted^10^. Particularly, it has been commonly agreed that observer design is critically important for all feedback control designs in terms of state space methodologies^11^. For model-free observers (MFO), which means designing the observers without using the system model, only measured input and output, and the system dynamic order are used to fit some pre-defined observers by tuning the gains of the state estimation errors. There have had various MFO configurations, such as sliding model observer^12^, high gain observers^10^, ADRC^3,13–15^. Conventional observers consider system state reconstruction. Extended state observer (ESO) estimates state as well external disturbance, further it can also estimate unknown model's perturbation^13^. One of the important applications of the ESO is associated with active disturbance rejection control (ADRC) which does not need the precise mathematical model of the plant. Therefore, ADRC is sometimes viewed as a type of complete model-free control. While only seeking state estimation, the ESO could be tailored in some way.

### U-model based control (U-control) system design

It has been a framework to separate plant dynamic inversion and controller design with configuration of a double feedback loop formulation^16–18^. The kernel of the U-control is the inner loop to cancel both plant nonlinearity and dynamics together so that the plant is converted into an identify matrix/a unit constant^19^. Then various control system designs can be effectively concisely implemented without involving plant models. Some of the U-control bench test applications have been reported ^20–22^.

### The major contribution of the study

In comparison with the other MFSMC approaches, with the author’s best knowledge, it could include.

1. This CMFSMC takes all types of systems/models as a bounded uncertainty. Therefore, it includes all the other partial MFSMC approaches as its special cases which require additional assumptions such as system model structures, unitary input gain^7^, state measurable^23^, extra effort to deal with chattering effect, based on discrete time approaches^5^. Further the novelty lays in the introduction of two equalities to assign the derivative of the sliding functions, which generally bridges the designs of those model-based SMC and model-free SMC.
2. This CMFSMC, integrated with U-control^16,18^, provides a robust dynamic/nonlinearity inversion scheme with a double sliding mode control, cancels both dynamics and nonlinearities together (in contract to feedback linearisation approach to cancel nonlinearities first, and then make coordinate transform into a linear system to design control, finally re-convert back to original control input^24^. Accordingly it makes the total system configurated into a double loop control system with both state feedback and output feedback, that is, using the inert loop (state feedback) with the CMFSMC for dynamic/nonlinear cancellation into an identity matrix or a unit constant, using the outer loop (output feedback) to design linear control system with an invariant controller to provide (1) the total closed loop control system performance and (2) the desired state vector for inner loop dynamic inversion.
3. Justify a robust model free state observer to provide the state estimate from the measured input and output.
4. With the CMFSMC, the inner and outer loop designs are separated and once off for all the systems satisfying the CMFSMC conditions.
5. In this study, Complete Mode-Free Sliding Mode Control means SMC design for dynamic inversion is model free, state estimate is model free, and further the whole control system design is model free because the plant dynamic/nonlinearity has been cancelled into an identity matrix.
6. Simulated case studies are provided to demonstrate the developed framework and show off the transparent design procedure for applications and expansions.

For the rest of the study, “Preliminary” introduces foundations for the follow up technical development. Section “CMFSMC” derives both DSMC and LESO, and proves/analyses the associated properties. Section "Model free U-control system design" presents a U-control framework to integrate all functional components into a CMFSMC. Section "Case studies" provides case studies for the computational experiments to demonstrate the analytical derivations. In addition, it is intended to provide a user transparent procedure for potential applications and expansions. Section "Conclusions" concludes the study.

## Preliminary

### Model based sliding mode control

Consider a general class of *n*th order single input dynamic system of

1 $$\dot X=F\left(X,u,d\right)$$

where $$X={\left[\begin{array}{cc}\begin{array}{cc}{x}_{1}& {x}_{2}\end{array}& \begin{array}{cc}\cdots & {x}_{n}\end{array}\end{array}\right]}^{T}\in {\mathbb{R}}^{n}$$ is the state vector, $u\in {\mathbb{R}}$ is the control input, and $d\in {\mathbb{R}}$ is a bounded unknown uncertainty. $F$, a function vector of the state $X$, input $u$, and uncertainty $d$ over a field $F, F\times F\to F$, is the operator mapping the underlying state, input, and uncertainty into the condensed expressions. For achieving SMC, the system states must be completely observable and controllable.

Let the desired state vector as ${X}_{d}=\left[{x}_{d},{\dot{x}}_{d}\cdots {x}_{d}^{\left(n-1\right)}\right]$, define a $\left(n-1\right)th$ order of state tracking error vector

2 $$E=X-{X}_{d}={\left[\begin{array}{ccc}e={x}_{1}-{x}_{d}& \dot{e}={x}_{2}-{\dot{x}}_{d}& \begin{array}{cc}\cdots & {e}^{\left(n-1\right)}={x}_{\left(n-1\right)}-{x}_{d}^{\left(n-1\right)}\end{array}\end{array}\right]}^{T}$$

Then set up a typical sliding function $S$^25^ in form of

3 $$S={c}_{1}e+{c}_{2}\dot{e}+\dots +{c}_{n-2}{e}^{\left(n-2\right)}+{e}^{\left(n-1\right)}$$

where the coefficient vector $$C=\left[\begin{array}{ccc}{c}_{1}& {c}_{2}& \begin{array}{cc}\cdots & {c}_{n-2}\end{array}\end{array}\right]\in {\mathbb{R}}_{\ge 0}$$ is chosen in terms of Hurwitz stable.

The other general type for assigning the sliding function^24^ can be expressed as

4 $$S={\left(\frac{d}{dt}+\lambda \right)}^{n-1}e$$

where $\lambda \in {\mathbb{R}}_{+}$ is the slop of the sliding function, a strictly positive constant to make the sliding function Hurwitz stable.

There have been many approaches for designing model-based SMC systems, for example^24^, the step by step procedure is shown below.

1. Define a sliding function $S$ for the error between the system state and desired reference state, which establishes a foundation for designing SMC to drive the states to and keep on the sliding surface in terms of $S=0.$
2. Set the derivative of the sliding surface to generate $\dot{S}=0$.
3. Derive the equivalent controller, ${u}_{eq}$ through $\dot{S}=0$ in conjunction with the plant nominal model.
  ${x}^{\left(n\right)}=f\left(x,u\right)$ and the pre-set desired reference state vector ${X}_{d}$.
4. To deal with the uncertainty, design the switch controller ${u}_{sw}$, by determining a discontinue control gain to satisfy the Lyapunov stability conditions $$\begin{array}{cc}V\left(*\right)=\frac{1}{2}{S}^{T}S>0,& \dot{V}\left(*\right)\end{array}<0, \forall \left(*\right)\ne 0$$ to attract states to the sliding surface $S=0$ and remain on the surface once arrived.
5. Finally formulate the SM controller as $u={u}_{eq}+{u}_{sw}$.

### Model based state observer

Consider a general single input and single output (SISO) state space model for uncertain nonlinear system below

5 $$\begin{array}{c}\dot{X}=F\left(X,u,d\right)=AX+B\left(u,X,d\right)\\ y=CX\\ X\left(0\right)={X}_{0}\end{array}$$

where $X\in {\mathbb{R}}^{n}$ for state vector, ${X}_{0}$ for initial state, $u\in {\mathbb{R}}$ and $y\in {\mathbb{R}}$ are the input and measurable output respectively, $d$ for the model uncertainty. $F=$, a function vector of the state $X$, input $u$, and uncertainty $d$ over a field $F, F\times F\to F$, is the operator mapping the underlying state, input, and uncertainty into the condensed expressions. $A\in {\mathbb{R}}^{n*n}$ for transition matrix, $B\in {\mathbb{R}}^{n}$ for the remaining part of $F-AX$, and $C\in {\mathbb{R}}^{1*n}$ for the output gain vector.

For state estimation, a general state observer^26^ can be configurated as

6 $$\dot{\widehat{X}}=A\widehat{X}+\mathcal{L}\left(y-C\widehat{X}\right)$$

where $\widehat{X}\in {\mathbb{R}}^{n}$ for the estimation of system state, $\mathcal{L}\in {\mathbb{R}}^{n}$ for the observer’s gain vector which is to be designed.

Define the observer estimated error vector $\tilde{X }=X-\widehat{X}$ to formulate the error dynamic equation as

7 $$\dot{\tilde{X }}=\left(A-\mathcal{L}C\right)\tilde{X }+B$$

The error equation provides a mechanism to design the observer’s gain $\mathcal{L}$, which properly drives the estimation error converged asymptotically.

### U-control (dynamic inversion and invariant controller)

The U-model based control (U-control in short) has two aspects, U-model to facilitate dynamic inversion and cancelation of nonlinearities, and U-control system design to provide a concise framework to implement control performance specifications in form of feedback and plant dynamic inversion, which the control performance also forms a desired state vector.

#### U-model and its dynamic inversion

A general SISO U-polynomial-model of $P$ ([16]), a mapping $u\to y,$ with a triplet of $\left(y\left(t\right),u\left(t\right),\alpha \left(t\right)\right)$, $y\left(t\right)\in {\mathbb{R}}$,$u\left(t\right)\in {\mathbb{R}},$ $\alpha \left(t\right)\in {\mathbb{R}}^{J}$ for a time variant parameter vector respectively at time $t\in {\mathbb{R}}^{+}$, is defined for describing dynamic plants as

8 $${y}^{\left(M\right)}={\mathcal{A}}^{T}\mathcal{U}=\sum_{j=0}^{J}{\alpha }_{j}{f}_{j}\left({u}^{\left(N\right)}\right), M\ge N$$

where ${y}^{\left(M\right)}$ and ${u}^{\left(N\right)}$ denote the $Mth$ and $Nth$ order derivatives of the output $y$ and input $u$ respectively. $J\in {\mathbb{R}}^{+}$ is the number of the polynomial terms. The time-varying parameter ${\alpha }_{j}\in {\mathbb{R}}$ is an absorbing function to include the other outputs $\left[{y}^{\left(M-1\right)},\dots ,y\right]\in {\mathbb{R}}^{M}$ and inputs $\left[{u}^{\left(N-1\right)},\dots ,u\right]\in {\mathbb{R}}^{N}$. ${f}_{j}\left(*\right)$ is a function of the input ${u}^{\left(N\right)}$. Vectors $${\mathcal{A}}^{T}=\left[\begin{array}{ccc}{\alpha }_{0}& ,\dots ,& {\alpha }_{J}\end{array}\right]$$ and $$\mathcal{U}={\left[\begin{array}{ccc}{f}_{0}& ,\dots ,& {f}_{J}\end{array}\right]}^{T}$$ over a field $F, F\times F\to F$ are the operators mapping the underlying input, output, and parameters into the condensed expressions.

##### *Remark 2.1*

In common, those exiting conventional models can be realised with U-model structure. In difference, U-model provides a unilateral control-oriented structure for cancellation of both nonlinearities and dynamics in one formulation^19^, which generally makes linear control system design approaches straightforwardly applicable to nonlinear systems no matter in forms of polynomial or state space models.

##### *Remark 2.2*

The polynomial U-model structure has been expanded to include state space models^27^, rational models^17^, and neural networks^28^ to facilitate dynamic inversions and the follow up U-control system designs.

Regarding U-model based dynamic inversion (UMDI), let U-model $P$, in forms of polynomial, be a mapping/function, $u\to y$. Then the UMDI $y\to u$ is a process of solution of its inverse ${P}^{-1}$, which can be generally expressed as

9 $${P}^{-1}\Leftrightarrow {u}^{\left(N\right)}\in {y}_{d}^{\left(M\right)}-\sum_{j=0}^{J}{\alpha }_{j}{f}_{j}\left({u}^{\left(N\right)}\right)={y}_{d}^{\left(M\right)}-{\mathcal{A}}^{T} \mathcal{U}=0, M\ge N$$

where ${y}_{d}^{\left(M\right)}$ is the specified desired output. Accordingly, the inverse of the model ${P}^{-1}$, a map from output to input, $y\to u$ is the solution of ${u}^{\left(N\right)}$ from the equation.

##### *Remark 2.3*

For the higher order derivative ${y}_{d}^{\left(M\right)}$ in UMDI, it can be determined in conjunction with invariant controller design in the U-control systems^19^.

#### U-control system design

Take $P$ for a general model describing dynamic plants which have properties as those frequently assumed in the many research works^29^.

1. The inverse ${P}^{-1}$ exists.
2. Lipschitz continuity satisfied, model $P$ is a mapping/function, $u\to y$, and its inverse ${P}^{-1}$ are diffeomorphic and globally uniformly Lipschitz in ${\mathbb{R}}$; that is,
  $$\Vert P\left({u}_{1}\right)-P\left({u}_{2}\right)\Vert \le {\gamma }_{1}P\Vert {u}_{1}-{u}_{2}\Vert , \forall {u}_{1},{u}_{2}\in {\mathbb{R}}$$
  $$\Vert {P}^{-1}\left({u}_{1}\right)-{P}^{-1}\left({u}_{2}\right)\Vert \le {\gamma }_{2}{P}^{-1}\Vert {u}_{1}-{u}_{2}\Vert , \forall {u}_{1},{u}_{2}\in {\mathbb{R}}$$

where ${u}_{1},{u}_{2}$ are the inputs while $P$ in form of polynomial model and replaced with states ${x}_{1},{x}_{2}$ while $P$ is a state equation, ${\gamma }_{1 }$ and ${\gamma }_{2}$ are the Lipschitz constants.

The U-control system is functionally expressed as

10 $$\sum =\left(\mathcal{F},\mathcal{C}\left({C}_{1},{P}^{-1}\right),P\right)\Leftrightarrow \sum =\left(\mathcal{F},{C}_{1},\mathcal{C}\left({P}^{-1},P\right)\right)\Leftrightarrow \sum =\left(\mathcal{F},{C}_{1},{I}_{ip}\right)$$

where $\mathcal{F}$ is the U-control system configuration, $\mathcal{C}\left(*\right)$ is a set of controllers, ${C}_{1}$ is a linear invariant controller, and ${I}_{ip}=\mathcal{C}\left({P}^{-1},P\right)$ is a unit constant or identity matrix.

**Figure 1:**
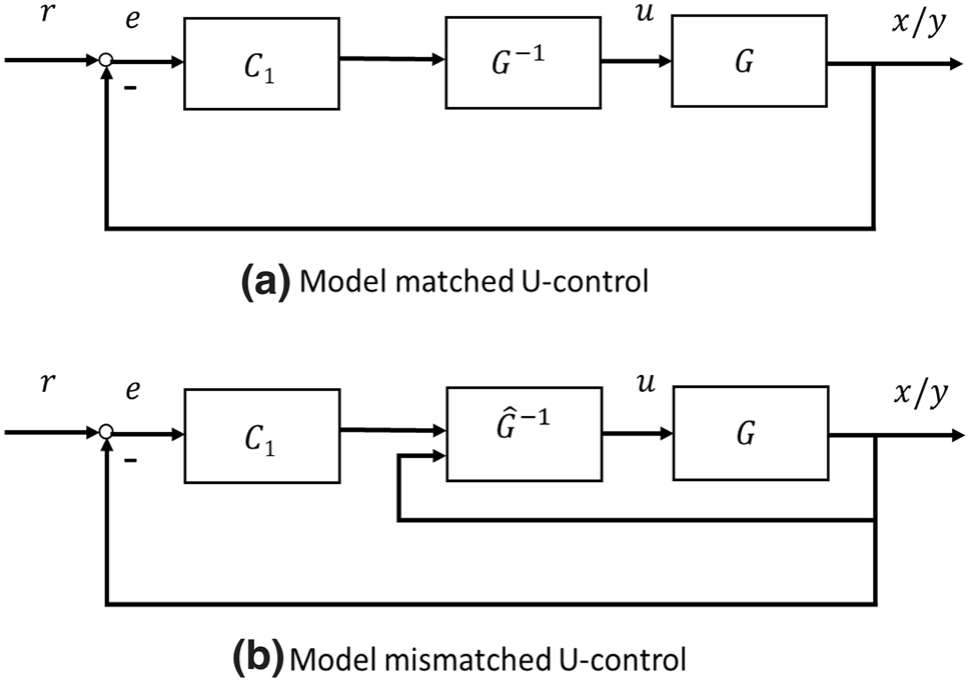
U-control systems.

Figure 1 shows model matched and model mismatched U-control configurations.

##### *Remark 2.4*

The U-control platform is unilaterally applicable to a wide range of dynamic systems while the dynamic inverse ${P}^{-1}$ exist^17,19^.

The step by step design procedure for Fig. 1a is listed below ([18]).

1. Design the dynamic inverter ${P}^{-1}$ to achieve $\mathcal{C}\left({P}^{-1},P\right)={I}_{ip}$, which gives $\sum =\left(\mathcal{F},{C}_{1},{I}_{ip}\right)$.
2. Design the invariant controller ${C}_{1}$ under $\sum =\left(\mathcal{F},{C}_{1},{I}_{ip}\right)$ with a required linear transfer function $G$, which gives ${C}_{1}=\frac{G}{1-G}$ in a closed loop configuration.
3. For generating the desired higher order output derivative ${y}_{d}^{\left(M\right)}$ or the desired state vector ${X}_{d}$, multiply a high-order filter ${F}_{1}=\frac{{s}^{M}}{{\left(\alpha s+1\right)}^{T}}, T\ge M,$ where $\frac{1}{\alpha }\gg {p}_{d}>0$, ${p}_{d}$ is the real part of the system dominant pole.

Figure 1b is an illustrative control system configuration, which will be expanded in the following sections.

## CMFSMC

This section involves in two aspects, design of SMC and observer.

### MFSMC

Consider a general SISO states space model for describing nonlinear dynamic systems

11 $$\dot{X}=F\left(X,u,d\right)$$

where $$X={\left[\begin{array}{cc}\begin{array}{cc}{x}_{1}& {x}_{2}\end{array}& \begin{array}{cc}\cdots & {x}_{n}\end{array}\end{array}\right]}^{T}\in {\mathbb{R}}^{n}$$ is the state vector, $u\in {\mathbb{R}}$ is the control input, and $d\in {\mathbb{R}}$ is bounded unknown external disturbance, and $F$ is a bounded unknown smooth nonaffine nonlinear vector function of the state vector $X$, the control input $u$, and the disturbance $d$. In this study $F$ is bounded but assumed unknown as total uncertainty^13^.

#### *Remark 3.1*

To formulate MFSMC, a straightforward view of the conventional model-based SMC is that the switching control has been already in somewhat of model free control, even though still using the bound of nominal model plus the uncertainty. For the equivalent control, the MFSMC requires some way to remove the design from the dependence of the nominal model. This study proposes a double sliding mode control (DSMC) approach to achieve the aim of the MFSMC.

For designing the MFSMC, define the same error vector as in Eq. (2)

12 $$E=X-{X}_{d}={\left[\begin{array}{ccc}e={x}_{1}-{x}_{d}& \dot{e}={x}_{2}-{\dot{x}}_{d}& \begin{array}{cc}\cdots & {e}^{\left(n-1\right)}={x}_{\left(n-1\right)}-{x}_{d}^{\left(n-1\right)}\end{array}\end{array}\right]}^{T}$$

Accordingly, assign a basis sliding function

13 $$S={c}_{1}e+{c}_{2}\dot{e}+\dots +{c}_{n-2}{e}^{\left(n-2\right)}+{e}^{\left(n-1\right)}$$

where coefficient vector $$C=\left[\begin{array}{ccc}{c}_{1}& {c}_{2}& \begin{array}{cc}\cdots & {c}_{n-2}\end{array}\end{array}\right]\in {\mathbb{R}}_{\ge 0}$$ is chosen in terms of Hurwitz stable.

The corresponding derivative of the sliding function is given by

14 $$\dot{S}={c}_{1}\dot{e}+{c}_{2}\ddot{e}+\dots +{c}_{n-2}{e}^{\left(n-1\right)}+{e}^{\left(n\right)}$$

Now assign two sliding functions for banded sliding surface and lined sliding surface respectively, which establishes a model-free SMC platform.

Global sliding banded function is specified with

15 $${S}_{g}=S+{\delta }_{1}, 0\le \left|{\delta }_{1}\right|\le \left|\delta \right|$$

where the sliding band function with thickness $\delta \ne 0$ is introduced.

Local sliding line function is specified with

16 $${S}_{l}=S+{\delta }_{2}, \left|{\delta }_{2}\right|=\left|\delta \right|=0$$

where the sliding line function with thickness $\delta =0$ is introduced. This band thickness approach neighbouring the switching surface has been widely used in dealing with chattering effect^24^. This new approach, will be explained technically shortly, is to derive a solution of the equivalent control to smooth the classical switching control to the equivalent control without suddenly forcing the derivative of the sliding function to zero.

**Figure 2:**
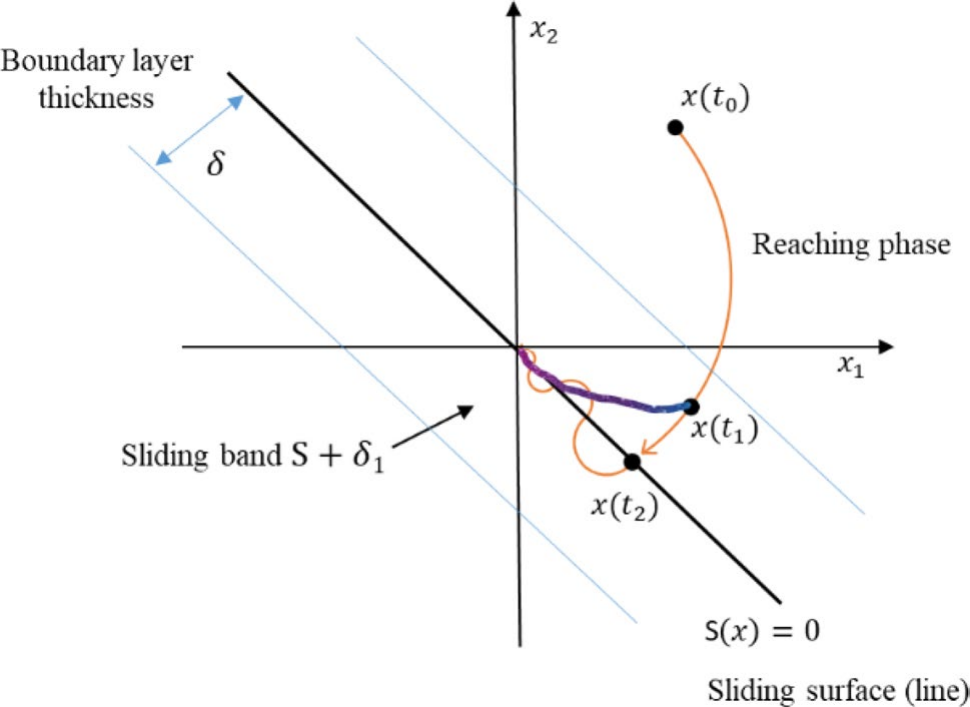
System states (red for classical SMC and red + blue for DSMC).

Figure 2 part of the figure from^30^ shows the double sliding mode control (DSMC) against the classical SMC.

For deriving the controllers, define switching control ${u}_{sw}$ and equivalent control ${u}_{sw}$ for attracting the states towards to the sliding band and sliding line respectively.

Define two Lyapunov functions, for ${V}_{g}$ for global and ${V}_{l}$ for local respectively, which are formulated below

17 $$\begin{array}{c}{V}_{g}={\frac{1}{2}\left({S}_{g}\right)}^{2}=\frac{1}{2}{\left(S+{\delta }_{1}\right)}^{2}\\ {V}_{l}={\frac{1}{2}\left({S}_{l}\right)}^{2}={\frac{1}{2}\left(S\right)}^{2}\end{array}$$

Accordingly, the derivatives of the Lyapunov functions are

18 $$\begin{array}{c}{\dot{V}}_{g}=\dot{S}\left(S+{\delta }_{1}\right)\\ {\dot{V}}_{l}=\dot{S}S\end{array}$$

#### Theorem 3.1

*There exit controllers* ${u}_{sw}=-{k}_{g}sgn\left(S+{\delta }_{1}\right)$ *and* ${u}_{eq}=-{k}_{l}S-\varepsilon$ *to make the MFSMC of BIBO system Eq.* (11) *asymptotically stable. The gains satisfy some proper conditions*.

#### *Proof*

**Figure 3:**
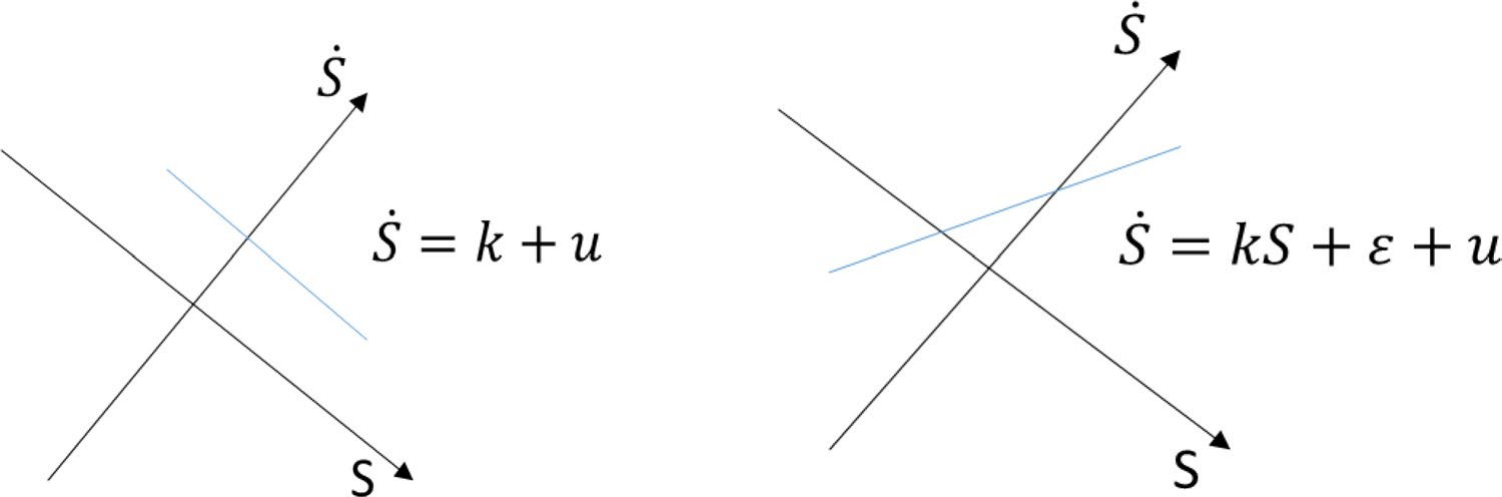
Sliding function coordinate system of ($S,\dot{S}$).

Introduce a sliding function coordinate system of ($S,\dot{S}$) to map the system to be controlled onto the sliding function plan for facilitating derivation of the controllers, two typical relationships are shown in Fig. 3

19 $$\begin{array}{c}\dot{S}=k+u\\ \dot{S}=kS+\varepsilon +u\end{array}$$

where $k=\left(X,u,d,{X}_{d}\right)$ and $\varepsilon$ is an offset of $kS$.

The first equation provides a bang–bang control to drive state to the convergent domain in the minimum-time and the second equation drives state to the convergent domain along a smooth trajectory. For SMC, combining the two equations with a switching boundary layer $\delta$ gives

20 $$\dot{S}=\left\{\begin{array}{ll}k+u& \forall \left|S\right|\ge \left|\delta \right|\\ kS+\varepsilon +u& \forall \left|S\right|<\left|\delta \right|\end{array}\right.$$

**Figure 4:**
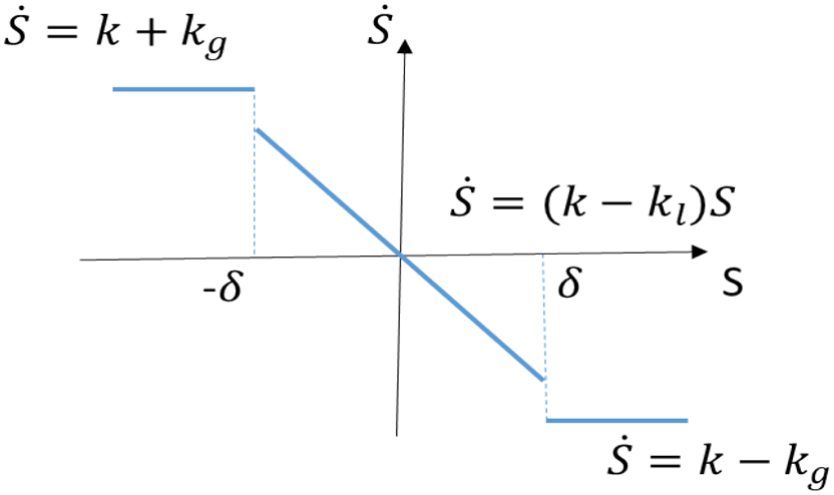
Combined sliding functions on ($S,\dot{S}$).

This is shown in Fig. 4.

Let the last line of system model Eq. (11) be

21 $${\dot{x}}_{n}={f}_{n}\left(X,u,d\right)+u$$

With reference to Eq. (20) and sliding function Eq. (13), the derivative of the sliding function can be expressed as

22 $$\dot{S}=\sum_{i=1}^{n-1}{c}_{1}{e}^{\left(i\right)}+{f}_{n}\left(X,u,d\right)+u-{x}_{d}^{\left(n\right)}=f+u$$

where $$f=\sum_{i=1}^{n-1}{c}_{1}{e}^{\left(i\right)}+{f}_{n}\left(X,u,d\right)-{x}_{d}^{\left(n\right)}$$

For SM switching control, let the derivative of sliding function Eq. (14) with

23 $$\dot{S}=\sum_{i=1}^{n-1}{c}_{1}{e}^{\left(i\right)}+{f}_{n}\left(X,u,d\right)+u-{x}_{d}^{\left(n\right)}=f+u=k+u$$

where $f=k=\sum_{i=1}^{n-1}{c}_{1}{e}^{\left(i\right)}+{f}_{n}\left(X,u,d\right)-{x}_{d}^{\left(n\right)}$, which satisfies $\mathrm{inf}\left(f\right)=m\le f\le \mathrm{sup}\left(f\right)=M,$ and $\left|m\right|\le \left|M\right|$. Assign the control

24 $${u=u}_{sw}=-{k}_{g}sgn\left(S+{\delta }_{1}\right)$$

where $sgn\left(*\right)$ is the signum function of the sliding surface $\left(S+{\delta }_{1}\right)$. Then substitute switching input into the derivative of the Lyapunov function Eq. (18) to give

25 $${\dot{{V}_{g}}=\dot{S}\left(S+{\delta }_{1}\right)=(f+u}_{sw})\left(S+{\delta }_{1}\right)=f\left(S+{\delta }_{1}\right)-{k}_{g}\left|S+{\delta }_{1}\right|$$

where ${k}_{g}$ is selected with ${k}_{g }\in {\mathbb{R}}_{>0}>\left|M\right|$. Therefore ${\dot{V}}_{g}\le 0$

For SM equivalent (smooth) control, assign the derivative of sliding function Eq. (14) with

26 $$\dot{S}=\sum_{i=1}^{n-1}{c}_{1}{e}^{\left(i\right)}+{f}_{n}\left(X,u,d\right)+u-{x}_{d}^{\left(n\right)}=f+u=kS+\varepsilon +u$$

where $$f=kS+\varepsilon =\sum_{i=1}^{n-1}{c}_{1}{e}^{\left(i\right)}+{f}_{n}\left(X,u,d\right)-{x}_{d}^{\left(n\right)}$$, and $$\mathrm{inf}\left(f\right)=m\le f=kS+\varepsilon \le \mathrm{sup}\left(f\right)=M$$.

Assign the control

27 $$u={u}_{eq}=-{k}_{l}S-\varepsilon$$

Then substitute it into the derivative of the Lyapunov function Eq. (18) to give

28 $${{\dot{V}}_{l}=\dot{S}S=(f+u}_{eq})S={(kS +u}_{eq})S=k{S}^{2}-{k}_{l}{S}^{2}$$

where $${k}_{l}\in {\mathbb{R}}_{>0}>\left|\mathrm{sup}\left(k\right)\right|$$. Therefore $${\dot{V}}_{l}\le 0$$.

Now, for the whole MFSMC, let the composite Lyapunov function be

29 $$V={V}_{g}+{V}_{l}$$

With the specified controls of ${u}_{sw}=-{k}_{g}sgn\left(S+{\delta }_{1}\right)$ and ${u}_{eq}=-{k}_{l}S$, the Lyapunov stability conditions can be proved with

30 $$\begin{array}{c}V={V}_{g}+{V}_{l}\ge 0\\ \dot{V}={\dot{V}}_{g}+{\dot{V}}_{l}\le 0\end{array}$$

#### QED

##### *Remark 3.2*

Lyapunov stability analysis have been used twice to derive the DSMC to achieve MFSMC. The DSMC design procedure actually is a process of the proof. The first Lyapunov stability ($$\begin{array}{cc}{V}_{g}\ge 0& {\dot{V}}_{g}\le 0\end{array}$$) used is to drive the state vector $x$ converged to the sliding mode band ${S}_{g}=S+{\delta }_{1}, 0\le \left|{\delta }_{1}\right|\le \left|\delta \right|$ by switching control. The second Lyapunov stability ($$\begin{array}{cc}{V}_{l}\ge 0& {\dot{V}}_{l}\le 0\end{array}$$) used is to drive the state vector $X$ in the sliding band converge asymptotically to the final sliding mode line ${S}_{l}=S+{\delta }_{2}=S, {\delta }_{2}=0$ by continuous equivalent control.

##### Theorem 3.2

*With the selection of* $\dot{S}=kS+\varepsilon +u=kS+{u}_{eq}={-(k}_{l}-k)S{|}_{{(k}_{l}-k)>0}+\varepsilon$, *the sliding function* $S-\frac{\varepsilon }{{(k}_{l}-k)}$ *monotonically exponentially converges to zero with the decay rate of* ${k}_{l}-k$.

##### *Proof*

The solution of the 1st order differential equation of $\dot{S}+{(k}_{l}-k)S=\varepsilon$ is $S\left(t\right)=\frac{\varepsilon }{{(k}_{l}-k)}\left(1-Exp\left({-(k}_{l}-k\right)t\right)$. Therefore $S\left(t\right)-\frac{\varepsilon }{{(k}_{l}-k)}=-\frac{\varepsilon }{{(k}_{l}-k)}Exp({-(k}_{l}-k)t)$ and $\underset{t\to \infty }{\mathrm{lim}}\left(S\left(t\right)-\frac{\varepsilon }{{(k}_{l}-k)}\right){|}_{{(k}_{l}-k)>0}\to 0$. The derivative of the sliding function $\dot{S}$ follows $\dot{S}=\varepsilon Exp\left({-(k}_{l}-k)t\right)$ and $\underset{t\to \infty }{\mathrm{lim}}\dot{S}=\underset{t\to \infty }{\mathrm{lim}}\varepsilon Exp({-(k}_{l}-k)t){|}_{{(k}_{l}-k)>0}\to 0$

#### QED

##### Theorem 3.3

*The selections of* $\dot{S}=kS+\varepsilon +u$ *and* $\dot{S}=k+u$ *present a generalisation of accurate model-based, nominal model-based, and model-unknown/free SMC by assigning the derivatives of the sliding functions with different expressions*.

##### *Proof*

The three cases are analysed below.

For accurate model-base, let $\dot{S}=kS+\varepsilon =f+u=0$, so that the equivalent control is determined by $u=-f$

For nominal model-based, $f$ is not exactly known, but assume it is bounded with $\left|\widehat{f}-f\right|\le F\left(X\right)$, where $\widehat{f}$ is the estimate of $f$. Let $\dot{S}=kS+\varepsilon =\widehat{f}+u=0$, consequently, $u=-\widehat{f}-ksgn\left(S\right)$, where, $k=F+\eta$ and $\eta$ is a strictly positive constant ([23]).

For model-unknown/free,$f$ is assumed bounded with $\mathrm{inf}\left(f\right)=m\le f\le \mathrm{sup}\left(f\right)=M$, let $\dot{S}=kS+\varepsilon +u$ and $u=-{k}_{l}S+\varepsilon -{k}_{g}sgn\left(S+{\delta }_{1}\right)$, where ${k}_{l}\in {\mathbb{R}}_{>0}>\left|\mathrm{sup}\left(k\right)\right|$ and ${k}_{g }\in {\mathbb{R}}_{>0}>\left|M\right|$. It should be noted that in this case, it cannot determine control $u$ by letting the derivative of sliding function $\dot{S}=0$. Alternatively, applying twice of Lyapunov stability theorem to determine the global switching gain and local smooth gain.

#### QED

##### *Remark 3.3*

Comparison of the designs between model-based and model free shows that model-based approaches use the derivative of the sliding function $\dot{S}=0$ to determine the control $u$, this model-free approach takes up twice of Lyapunov stability theorem to determine the global switching gain and local smooth gain to satisfy $V={V}_{g}+{V}_{l}\ge 0$ and $\dot{V}={\dot{V}}_{g}+{\dot{V}}_{l}\le 0$.

### Stats observer

For a class of SISO nonlinear system of $y^{\left( n \right)} = f\left( {y^{{\left( {n - 1} \right)}} , \ldots y,u,d} \right)$, where $f\left(*\right)$ representing the nonlinear dynamics and has proper properties (dynamic plant invertible, state observable/controllable, stabilisable, stable zero dynamics), $d$ is the external disturbance. By letting $$\begin{array}{ccc}y={x}_{1}& \begin{array}{cc}{y}^{\left(1\right)}={x}_{2}={\dot{x}}_{1}& \cdots \end{array}& {y}^{\left(n\right)}={\dot{x}}_{n}\end{array}$$, A generalised triangle state space model for realising the nonlinear system input/output relationship can be determined below

31 $$\begin{array}{c}\begin{array}{c}{\dot{x}}_{1}={x}_{2}\\ \vdots \end{array}\\ {\dot{x}}_{n-1}={x}_{n}\\ \begin{array}{c}{\dot{x}}_{n}=f\left(X,u,d\right)+u\\ \\ y={x}_{1}\end{array}\end{array}$$

where state vector $$X={\left[\begin{array}{ccc}{x}_{1}& \cdots & {x}_{n}\end{array}\right]}^{T}\in {\mathbb{R}}^{n}$$.

#### *Remark 3.4*

There is a one-to-many relationship between an input/output model and minimal state space realisations because many state-space realisations can produce the same input/output behaviour. Accordingly, assume such transforms exist for the conversions of the state models while keeping the consistence with the system input–output behaviour. By such assumption, this study proposed state space model and its observer imply the input/output equivalence with the other state space models and the ad hoc observers.

In this study, a linear extended state observer (LESO)^13^ is adopted for reconstructing the state variables for the consequent state space model-free control system design.

The LESO is given below

32 $$\begin{array}{*{20}c} {\mathop {\hat{x}}\limits^{ \cdot }_{i} = \hat{x}_{i + 1} + \omega_{o}^{i} \alpha_{i} \left( {y - \hat{x}_{1} } \right),\quad i = 1,2, \ldots n - 1} \\ \vdots \\ {\mathop {\hat{x}}\limits^{ \cdot }_{n} = \hat{x}_{n + 1} + \omega_{o}^{n} \alpha_{n} \left( {y - \hat{x}_{1} } \right) + u} \\ {\mathop {\hat{x}}\limits^{ \cdot }_{n + 1} = \omega_{o}^{n + 1} \alpha_{n + 1} \left( {y - \hat{x}_{1} } \right)} \\ \end{array}$$

where ${\omega }_{o}>0$ is the observer bandwidth, normally assigned by system bandwidth or trial and error approach in advance. ${\alpha }_{i}\in 1 \cdots n+1$ are the regulable constants to satisfy the Hurwitz stability condition and generally, determined by^31^

33 $${\left(s+1\right)}^{n+1}={s}^{n+1}+{\alpha }_{1}{s}^{n}\cdots +{\alpha }_{n}s+{\alpha }_{n+1}$$

where $$\alpha_{i} = \frac{{\left( {n + 1} \right)!}}{{i!\left( {n + 1 - i} \right)!}}, i = 1, 2, \ldots n + 1.$$

#### *Remark 3.5*

The analysis of this type of LESO^31^ covers two cases of (1) the system input/output model $f\left(*\right)$ known and (2) the system model $f\left(*\right)$ is unknown. It has been proved^31^ that

1. For a known input/output function $f\left(*\right)$, the estimation errors are converged with
  $\mathop {{\text{lim}}}\limits_{t \to \infty } \tilde{x}_{i} \left( t \right) = 0,{ }i = 1,2, \ldots n + 1$ where the estimation errors are defined with ${\tilde{x }}_{i}={x}_{i}-{\widehat{x}}_{i}$.
2. For unknown input/output function $f\left(*\right)$, the state estimation error is bounded and its upper bound monotonously decreases with the observer bandwidth. It is noted that the second property provides a framework for model free state estimation.

#### *Remark 3.6*

The LESO shares the commonalities with high gain observers, which this type of observers provides a very natural platform for the state reconstruction, particularly effective while in the situation of lack knowledge on the system dynamics. Comprehensively, such observers can be appropriately integrated with state feedback to give output feedback^10^.

## Model free U-control system design

### The control objectives are summarised below

For a class of general dynamic systems given in Eq. (11), $\dot{X}=F\left(X,u,d\right)$, the aim of the U-control is to drive the model-unknown systems to track a desired reference trajectory in request, which is configurated by a robust state feedback dynamic/nonlinearity inversion and a robust output feedback trajectory tracking control. The major objectives include

1. Robust DSMC based dynamic/nonlinearity inversion.
2. LESO for state vector estimation.
3. Control system performance specification/implementation including desired state vector assignment.

**Figure 5:**
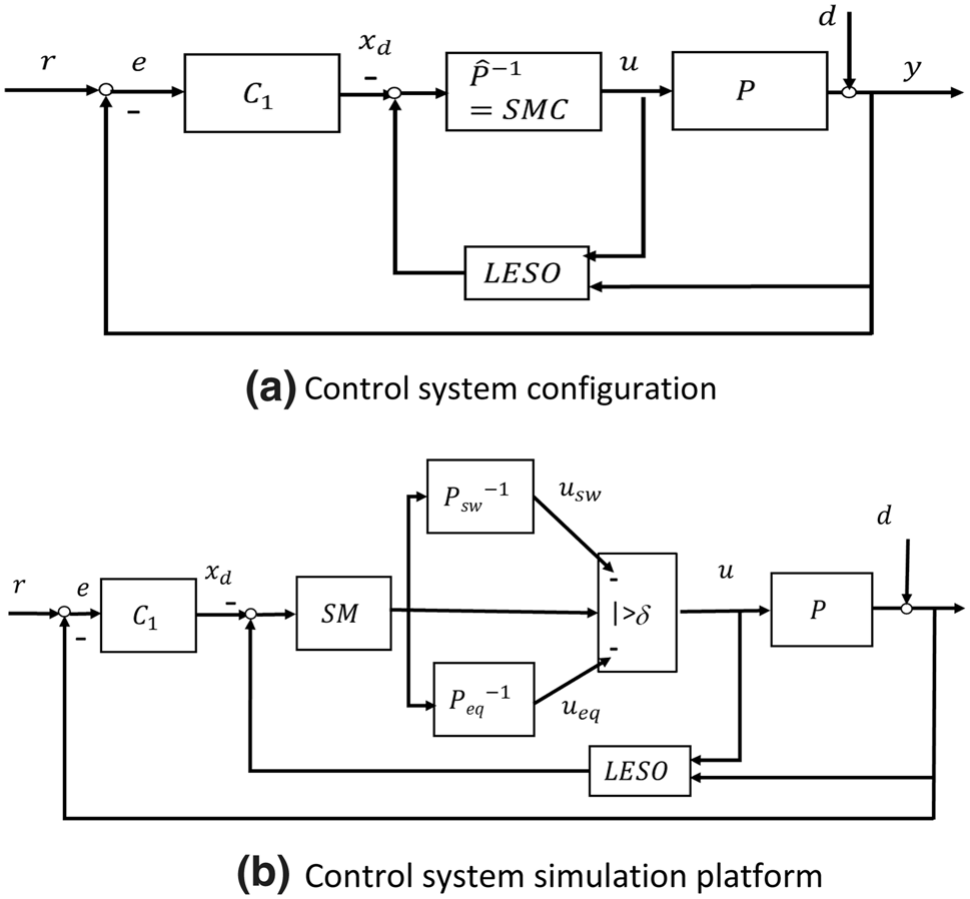
CMFSMC.

Figure 5 shows the model free U-control system with the configuration and the simulation platform.

Figure 5a is an illustrative schematic diagram. The step by step design procedure for Fig. 5b is listed below.

The main roles of the two loops in the control system configuration are briefly summarised below.

#### The inner loop

Plant model-fee dynamic/nonlinearity inversion includes DSMC and LESO which have developed. The rest of the parameters tuning include in $DSMC\left(S,\delta ,{k}_{g},{k}_{l}\right)$ and $LESO\left({\omega }_{o}, {\alpha }_{i},i=1\cdots n+1\right)$, which the parameters have been defined in previous sections. In formulation of the SMC blocks in Fig. 5b, SM by Eq. (12), $\delta$ by trial and error or experience, ${k}_{g}{\in u}_{sw}$ by Eq. (24), ${k}_{l}{\in u}_{eq}$ by Eq. (27). In formulation LESO block in Fig. 5b, the bandwidth is assigned with ${\upomega }_{o}\ge \left(4\sim 5\right){\omega }_{s}$, where ${\omega }_{s}$ is the bandwidth of the sliding function, and ${\alpha }_{i}=1 \cdots n+1$ are determined by Eq. (33).

#### The external loop

For the external U-control loop, as explained in "U-control system design", (1) design the invariant controller ${C}_{1}$ under $\sum =\left(\mathcal{F},{C}_{1},{I}_{ip}\right)$ with a required linear transfer function $G$ to specify the whole system desired output and state responses, which gives ${C}_{1}=\frac{G}{1-G}$ in a closed loop configuration. (2) For generating the desired higher order output derivative ${y}_{d}^{\left(M\right)}$ or the desired state vector ${X}_{d}$, multiply a high-order filter ${F}_{1}=\frac{{s}^{M}}{{\left(\alpha s+1\right)}^{T}}, T\ge M,$ where $\frac{1}{\alpha }\gg {p}_{d}>0$, ${p}_{d}$ is the real part of the system dominant pole. The design details can be referred to U-control foundation work^19^.

##### *Remark 4.1*

Qualitatively, the stability conditions in the inner loop are determined by Lyapunov stability, which converges to an identity matrix monotonically exponentially (refer to Theorems 3.1 and 3.2) and the external loop designed is to satisfy the Hurwitz stability (obviously as the closed loop transfer function can be easily specified with assigning its all poles on the left half s plane. Accordingly, the whole control system is asymptotically stable with the designed structure and parameters.

##### *Remark 4.2*

While the LESO inserted in the control system, for the frequency bandwidth related parameters, it requires ${\upomega }_{o}\ge \left(4\sim 5\right){\omega }_{s}\ge \left(4\sim 5\right){\omega }_{n}$ to generate proper responses, where ${\upomega }_{o}$ is the LESO bandwidth, ${\omega }_{s}\mathrm{ is}$ the bandwidth of the sliding function, and ${\omega }_{n}$ is the bandwidth of the invariant controller. Many publications^13,31^ are available for the analysis of the LESO stability and convergence. This study is just borrowing the results in its control system configurations.

## Case studies

### Preparation

Two case studies are conducted for (1) investigating the numerical results with functionally configured control systems against those analytically derived, (2) illustrating the design process with a step by step procedure for potential applications and expansions.

The reference input is specified as a series of steps with

34 $$\begin{array}{cc}r\left(t\right)=\left\{\begin{array}{c}2\\ 6\\ \begin{array}{c}0\\ -2\end{array}\end{array}\right.& \begin{array}{c}2<t\le 10\\ 10<t\le 20\\ \begin{array}{c}20<t\le 30\\ 30<t\le 40\end{array}\end{array}\end{array}$$

A level external disturbance ($d\left(t\right)=1$) is added at each of the system output.

#### Control system design

This once off design is intended applicable for both of the cases in the simulation study.

#### DSMC

Set sliding function $S=20e+\dot{e}$. For each case, the control tasks are tuning the gains (${k}_{g},{k}_{l}$) and the sliding band thickness $\delta$

#### LESO

For both cases, assign the observer bandwidth ${\upomega }_{o}=100$ and the corresponding LESO is given below

35 $$\begin{aligned} & \mathop {\hat{x}}\limits^{ \cdot }_{1} = \hat{x}_{2} + \alpha_{1} \omega_{o} \left( {y - \hat{x}_{1} } \right) \\ & \mathop {\hat{x}}\limits^{ \cdot }_{2} = \hat{x}_{3} + \alpha_{2} \omega_{o}^{2} \left( {y - \hat{x}_{1} } \right) + u \\ & \mathop {\hat{x}}\limits^{ \cdot }_{3} = \alpha_{3} \omega_{o}^{3} \left( {y - \hat{x}_{1} } \right) \\ \end{aligned}$$

where $$\begin{array}{ccc}{\alpha }_{1}=3& {\alpha }_{2}=3& {\alpha }_{3}\end{array}=1$$, which are determined from $\frac{1}{{\left(s+{\omega }_{o}\right)}^{3}}=\frac{1}{{s}^{3}+3{s}^{2}+3s+{\omega }_{o}^{3}}=\frac{1}{{s}^{3}+{\alpha }_{1}{s}^{2}+{\alpha }_{2}s+{\alpha }_{3}{\omega }_{o}^{3}}$

#### U-control

For both cases, assign the desired closed loop transfer function with $G=\frac{{\omega }_{n}^{2}}{{s}^{2}+2\upzeta {\omega }_{n}s+{\omega }_{n}^{2}}$ with ${\omega }_{n}=5$ and $\upzeta =0.7$, that is, $G=\frac{25}{{s}^{2}+7s+25}$. Accordingly, the invariant controller ${C}_{1}=\frac{G}{1-G}=\frac{25}{{s}^{2}+7s}$. Consequently, the two desired states are assigned as $$\begin{array}{cc}{x}_{d1}=\frac{1}{s}\frac{25}{s+7},& {\dot{x}}_{d1}{=x}_{d2}=\frac{25}{s+7}\end{array}$$.

#### Assigned bandwidth

In summary, in the above design, it has assigned ${\omega }_{n}=5$ (the bandwidth of the invariant controller), ${\omega }_{s}=20$ (the bandwidth of the sliding function), and ${\upomega }_{o}=100$ (the LESO bandwidth).

### Simulation demonstrations

#### Case 1: Control of nonlinear non-affine dynamic system

The considered nonlinear dynamic system is expressed with

36 $${y}^{\left(2\right)}=-0.6{y}^{\left(1\right)}-y{y}^{\left(1\right)}-u{y}^{\left(1\right)}+\mathrm{sin}\left(u\right)+2u+{u}^{3}+d\left(t\right)$$

where $y, u$ are the system output and input respectively.

By letting $$\begin{array}{ccc}y={x}_{1}& {y}^{\left(1\right)}={x}_{2}={\dot{x}}_{1}& {y}^{\left(2\right)}={\dot{x}}_{2}\end{array}$$, the system state space realisation is determined in form of

37 $$\begin{aligned} & \dot{x}_{1} = x_{2} \\ & \dot{x}_{2} = - 0.6x_{2} - x_{1} x_{2} - ux_{2} + \sin \left( u \right) + 2u + u^{3} \\ & y = x_{1} + d\left( t \right) \\ \end{aligned}$$

By trial and error approach, the DSMC acting as the dynamic/nonlinearity inverter is tuned with the gains ${k}_{g}=-5$ and ${k}_{l}=-4$ and the sliding band thickness $\delta =1$

**Figure 6:**
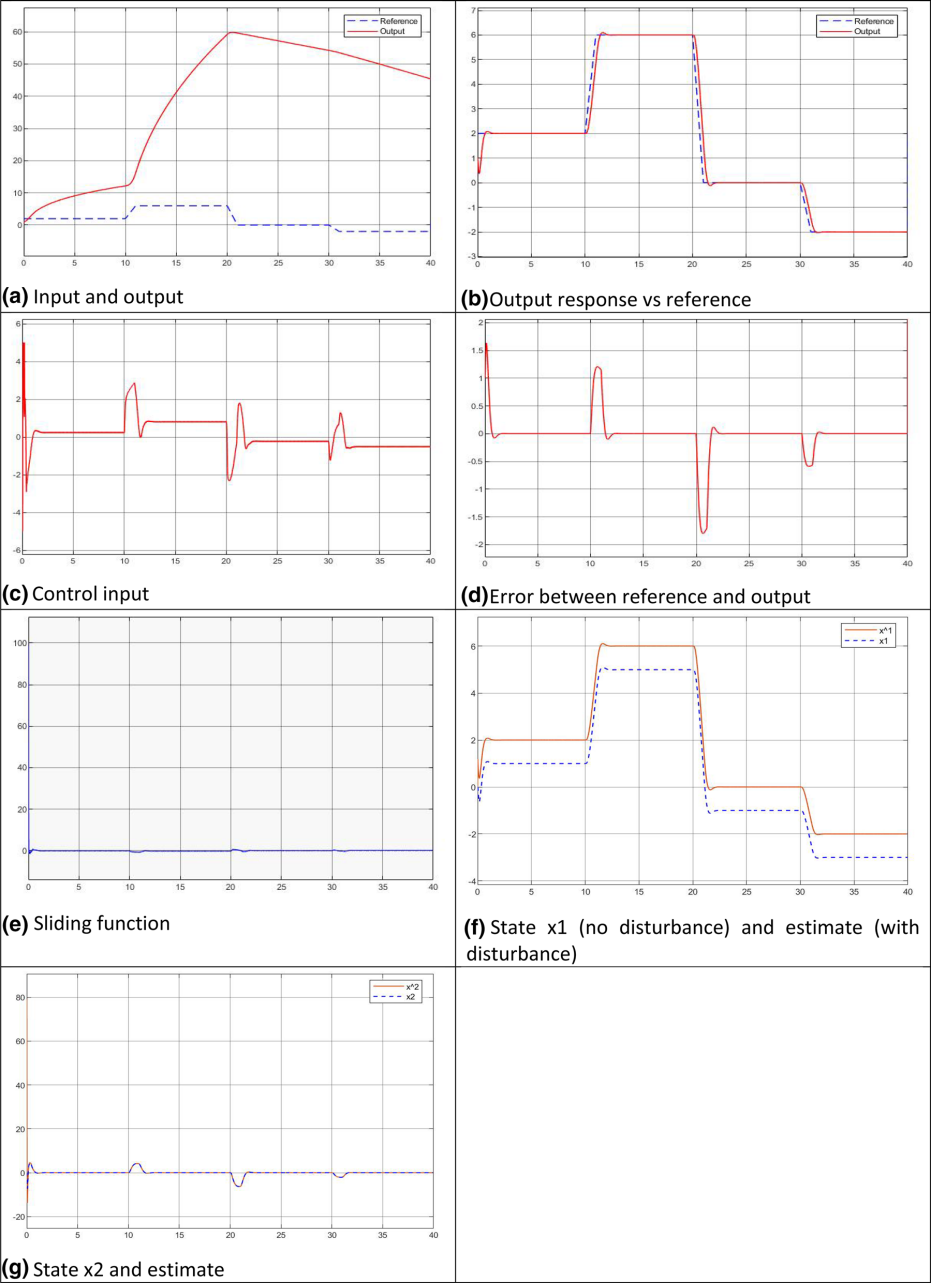
Case 1—simulated plots.

Figure 6 shows a pack of the generated plots.

#### Case 2: Control of Van de Pol (VDP) oscillator dynamics

Regarding the characteristics, the Van der Pol oscillator is a non-conservative system with nonlinear damping to follow a second-order dynamic^32^. For controlled VDP system, it has

38 $${y}^{\left(2\right)}=\mu \left(1-{y}^{2}\right){y}^{\left(1\right)}-y+u$$

where $y, u$ are the system output and input respectively.

By letting $$\begin{array}{ccc}y={x}_{1}& {y}^{\left(1\right)}={x}_{2}={\dot{x}}_{1}& {y}^{\left(2\right)}={\dot{x}}_{2}\end{array}$$, the VPD system state space realisation is determined in form of

39 $$\begin{aligned} & \dot{x}_{1} = x_{2} \\ & \dot{x}_{2} = \mu \left( {1 - x_{1}^{2} } \right)x_{2} - x_{1} + u \\ & y = x_{1} + d\left( t \right) \\ \end{aligned}$$

For this case study assign $\mu =1.5$, which is a parameter for the nonlinearity and the damping strength.

By trial and error approach, the DSMC acting as the dynamic/nonlinearity inverter is tuned with the gains ${k}_{g}=-100$ and ${k}_{l}=-50$ and the sliding band thickness $\delta =5$.

**Figure 7:**
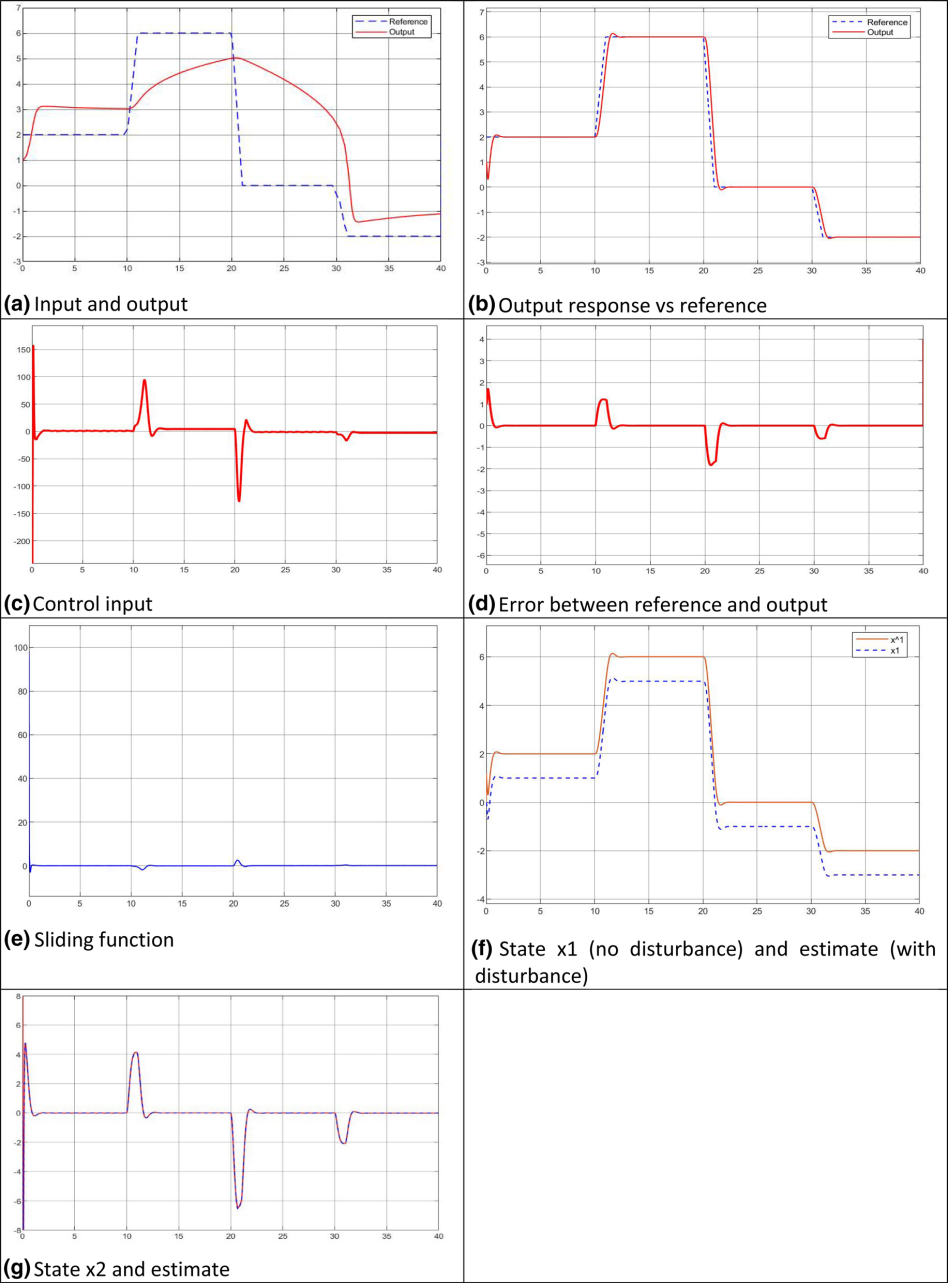
Case 2—simulated plots.

Figure 7 shows a pack of the generated plots.

### Discussions on the simulated results

1. The plant input/output relationships with the both cases are tested with the reference input sequence before the control systems built up, which indicate they are input/output bounded nonlinear dynamics.
2. The two gains and the sliding band thickness work well in the ranges of case 1 (${-6\le k}_{g}\le -4$, ${-7\le k}_{l}\le -3$, $0.6\le \delta \le 2$) and case 2 (${-150\le k}_{g}\le -100*$, ${-50\le k}_{l}\le -20$, $1\le \delta \le 5$).
3. The system outputs at both cases well follow the specified linear dynamic system performances (transient/steady state response, control input, and output errors).
4. Even with an external disturbance $d\left(t\right)=1$, the steady state errors between the reference and the output are zero. This is consistent with the analytically predicted steady state error, ${e}_{ss}=\underset{s\to 0}{\mathrm{lim}}\frac{s}{s}\frac{1+D\left(s\right)}{1+{C}_{1}}=\underset{s\to 0}{\mathrm{lim}}\frac{1+1}{1+{C}_{1}}=\frac{1+1}{1+\infty }=0$
5. The LESO works well for both cases. With the U-control, the simulations have demonstrated that comprehensively, such observers are appropriately integrated with state feedback to give output feedback^10^.
6. Both cases demonstrate that the sliding function $S-\frac{\varepsilon }{{(k}_{l}-k)}$ monotonically exponentially converges to zero with the decay rate of ${k}_{l}-k$.
7. This type of model-free/data driven control does not require conventional data iteration in the while process, this is because the use of twice Lyapunov stability condition guides the convergent direction and the gains designed generate possible power to drive the systems along the trajectories to the convergent states.
8. It should be noted that the difference of the state and its estimate in Figs. 6f and 7f is caused from the external constant disturbance with amplitude 1 $(d\left(t\right)=1$) referring to "Preparation", therefore the error = difference between the real state and estimated. Bear in mind, the state is not used for feedback control, just an indication in case of disturbance free. In the simulated control the estimated states are used for control feedback in representing the realistic situation with added disturbance. This well demonstrates the LESO performance to estimate both state and disturbance. Jointly the disturbance is dealt with the both inner loop and external loop. The figures, with the others simulated, demonstrate the expected control performance even with external disturbance.

Jointly the disturbance is dealt with the both inner loop and external loop. The figures, with the others simulated, demonstrate the expected control performance even with external disturbance.

## Conclusions

The study has taken a system to be controlled as an uncertainty, except assuming the system having some reasonably known characteristics, such satisfying Lipschitz conditions, bounded, controllable/observable, and dynamic order known. The once a control system performance specified, the rest of the DSMC controller tuning is to use trial and error to find the four parameters in the $DSMC\left(S,\delta ,{k}_{g},{k}_{l}\right)$.

DSMC plays a kernel role in the dynamic/nonlinearity inversion, therefore in the whole U-control system design. The novelty lays in the introduction of two equalities to assign the derivative of the sliding functions instead of just letting it be zero, which bridges the designs of those model-based SMC and model-free SMC. The by-product of the DSMC is to relieve the chattering effect without additional functions inserted in SMC design.

This is the first stage work on CMFSMC with focus on system configuration, functioning components, basic property analysis, and numerical validation with the integrated function blocks. The next stage study could be a rigour mathematical descriptions and proofs of some of the details.

Surely more critical bench tests are needed to find out drawbacks of the results for further improvement. The other potential study could be the expansion of the SISO procedure to the MIMO cases.

## References

[1] Hou ZS, Wang Z (2013). From model-based control to data-driven control: Survey, classification and perspective. Inf. Sci..

[2] Fliess M, Cédric Join C (2013). Model-free control. Int. J. Control.

[3] Han JQ (2009). From PID to active disturbance rejection control. IEEE Trans. Ind. Electron..

[4] Jiang Y, Jiang ZP (2015). Global adaptive dynamic programming for continuous-time nonlinear systems. IEEE Trans. Autom. Control.

[5] Crassidis, A. & Reis, R.M. Model-free sliding mode control method. in *Proceedings of the 3rd International Conference on Control, Dynamic Systems, and Robotics (CDSR’16), Ottawa, Canada*. Paper 100 (2016).

[6] Tin, F.E. A model-free control system based on the sliding mode control method with applications to multi-input-multi-output systems. PhD thesis, (Rochester Institute of Technology. Rochester, New York 2017).

[7] Precup RE, Radac MB, Roman RC, Petriu EM (2017). Model-free sliding mode control of nonlinear systems: Algorithms and experiments. Inf. Sci..

[8] Ebrahimi N, Ozgoli S, Ramezani A (2018). Model-free sliding mode control, theory and application. Proc. Inst. Mech. Eng. Part I J. Syst. Control Eng..

[9] Hou HZ (2021). Sliding mode control of networked control systems: An auxiliary matrices based approach. IEEE Trans. Autom. Control.

[10] Khalil HK, Praly L (2013). High-gain observers in nonlinear feedback control. Int. J. Robust Nonlinear Control.

[11] Tsui CC (2015). Observer design—A survey. Int. J. Autom. Comput..

[12] Chawengkrittayanont P, Pukdeboon C (2019). Continuous higher order sliding mode observers for a class of uncertain nonlinear systems. Trans. Inst. Meas. Control..

[13] Guo BZ, Zhao ZL (2011). On the convergence of an extended state observer for nonlinear systems with uncertainty. Syst. Control Lett..

[14] Radke, A. & Gao, Z.Q. A survey of state and disturbance observers for practitioners. in *American Control Conference*. Paper 9047129 (2006).

[15] Ding SH, Chen WH, Mei KQ, Murray-Smith DJ (2019). Disturbance observer design for nonlinear systems represented by input–output models. IEEE Trans. Ind. Electron..

[16] Zhu QM, Guo LZ (2002). A pole placement controller for non-linear dynamic plants. Proc. Inst. Mech. Eng. Part I J. Syst. Control Eng..

[17] Zhu QM, Liu L, Zhang WC, Li SY (2018). Control of complex nonlinear dynamic rational systems. Complexity.

[18] Zhang WC (2020). U-Model and U-control methodology for nonlinear dynamic systems. Complexity.

[19] Li RB, Zhu QM, Kiely J, Zhang WC (2020). Algorithms for U-model-based dynamic inversion (UM-dynamic inversion) for continuous time control systems. Complexity.

[20] Hussain NAA, Ali SSA, Ovinis M, Arshad M, Al-saggaf UM (2020). Underactuated coupled nonlinear adaptive control synthesis using U-model for multivariable unmanned marine robotics. IEEE Access.

[21] Yan H, Li JF, Nouri H, Xu LL (2020). U-model-based finite-time control for nonlinear valve-controlled hydraulic servosystem. Math. Probl. Eng..

[22] Wei W, Duan BW, Zuo M, Zhu QM (2021). An extended state observer based U-model control of the COVID-19. ISA Trans..

[23] Schulken, E. & Crassidis, A. Model-free sliding mode control algorithms including application to a real-world quadrotor. in *Proceedings of the 5th International Conference of Control, Dynamic Systems, and Robotics (CDSR'18), Niagara Falls, Canada—June 7–9, 2018*. Paper 112 (2018).

[24] Slotine JJE, Li WP (1991). Applied Nonlinear Control.

[25] Yan XG, Spurgeon SK, Edwards C, Yan X-G, Spurgeon SK, Edwards C (2017). Variable structure control of complex systems. Communications and Control Engineering.

[26] Liu Z, Karimi HR, Yu JP (2020). Passivity-based robust sliding mode synthesis for uncertain delayed stochastic systems via state observer. Automatica.

[27] Geng XP, Zhu QM, Liu T, Na J (2019). U-model based predictive control for nonlinear processes with input delay. J. Process Control.

[28] Zhu QM, Zhang WC, Zhang JH, Sun B (2019). U-neural network-enhanced control of nonlinear dynamic systems. Neurocomputing.

[29] Isidori A (2013). Nonlinear Control Systems.

[30] Yang I, Lee D, Han DS (2014). Designing a robust nonlinear dynamic inversion controller for spacecraft formation flying. Math. Probl. Eng..

[31] Zheng, Q., Gao, L.Q. & Gao, Z.Q. On stability analysis of active disturbance rejection control for, nonlinear time-varying plants with unknown dynamic. in *Proceedings of the 46th IEEE Conference on Decision and Control, New Orleans, LA, USA, paper-ThB07.6*. (2007).

[32] Wikipedia. Van der Pol Oscillator. https://en.wikipedia.org/wiki/Van_der_Pol_oscillator. (Accessed 9 August 2021).

